# Niagara County Medical Society, Old and New

**Published:** 1872-06

**Authors:** 


### Niagara County Medical Society, Old and New.

It appears from articles in the *Lockport Morning Times*, that the old Medical Society, organized in 1823, was declared in January, 1871, dissolved or disbanded. A new Society was then organized, new by-laws adopted, and officers duly chosen. It now appears that the Society will not remain in solution, and a call was made by twelve physicians of the county, members of the old society to meet at the Judson House Tuesday, June first. All regular physicians who have not done so, were invited to join the Society and take part in the election of officers.

